# Trade-offs between receptor modification and fitness drive host-bacteriophage co-evolution leading to phage extinction or co-existence

**DOI:** 10.1093/ismejo/wrae214

**Published:** 2024-10-23

**Authors:** Lin Chen, Xue Zhao, Shelyn Wongso, Zhuohui Lin, Siyun Wang

**Affiliations:** Food, Nutrition and Health, Faculty of Land and Food Systems, The University of British Columbia, 2205 East Mall, Vancouver, British Columbia V6T 1Z4, Canada; School of Chemistry, Chemical Engineering and Biotechnology, Nanyang Technological University, 62 Nanyang Drive, Singapore 637459, Singapore; Department of Biological Systems Engineering, Virginia Tech, 1230 Washington Street SW, Blacksburg, Virginia 24061, United States; Food, Nutrition and Health, Faculty of Land and Food Systems, The University of British Columbia, 2205 East Mall, Vancouver, British Columbia V6T 1Z4, Canada; Department of Microbiology and Immunology, The University of British Columbia, 2350 Health Sciences Mall, Vancouver, British Columbia V6T 1Z3, Canada; Food, Nutrition and Health, Faculty of Land and Food Systems, The University of British Columbia, 2205 East Mall, Vancouver, British Columbia V6T 1Z4, Canada

**Keywords:** co-evolution, co-existence, phage extinction, fitness, phage resistance

## Abstract

Parasite–host co-evolution results in population extinction or co-existence, yet the factors driving these distinct outcomes remain elusive. In this study, *Salmonella* strains were individually co-evolved with the lytic phage SF1 for 30 days, resulting in phage extinction or co-existence. We conducted a systematic investigation into the phenotypic and genetic dynamics of evolved host cells and phages to elucidate the evolutionary mechanisms. Throughout co-evolution, host cells displayed diverse phage resistance patterns: sensitivity, partial resistance, and complete resistance, to wild-type phage. Moreover, phage resistance strength showed a robust linear correlation with phage adsorption, suggesting that surface modification-mediated phage attachment predominates as the resistance mechanism in evolved bacterial populations. Additionally, bacterial isolates eliminating phages exhibited higher mutation rates and lower fitness costs in developing resistance compared to those leading to co-existence. Phage resistance genes were classified into two categories: key mutations, characterized by nonsense/frameshift mutations in *rfaH*-regulated *rfb* genes, leading to the removal of the receptor O-antigen; and secondary mutations, which involve less critical modifications, such as fimbrial synthesis and tRNA modification. The accumulation of secondary mutations resulted in partial and complete resistance, which could be overcome by evolved phages, whereas key mutations conferred undefeatable complete resistance by deleting receptors. In conclusion, higher key mutation frequencies with lower fitness costs promised strong resistance and eventual phage extinction, whereas deficiencies in fitness cost, mutation rate, and key mutation led to co-existence. Our findings reveal the distinct population dynamics and evolutionary trade-offs of phage resistance during co-evolution, thereby deepening our understanding of microbial interactions.

## Introduction

The age-old conflict between bacteriophages (phages) and their bacterial hosts profoundly shapes natural microbial ecosystems [1–4]. Additionally, with the increasing use of phages in medicine, veterinary, food, and biotechnology, the understanding of host-phage interactions is becoming increasingly important [5, 6]. An enigma surrounding host-phage interactions is the variety of co-evolutionary outcomes (e.g., population extinction and co-existence). It is believed that bacteria have larger genome sizes, providing them with greater evolutionary potential compared to their parasites [7–11]. This leads to the expectation that phage populations would become extinct in the face of multiple host defenses. Although such expectation holds true, phages co-exist with their hosts in many circumstances, both in laboratory and natural conditions [12–15]. These phenomena motivate the search for mechanisms to elucidate the dynamics and eventual cessation of host-phage co-evolution. Previous studies have explained this intriguing phenomenon by proposing the concept of fitness trade-offs [16–18]. In evolutionary biology, a prevalent paradigm suggests that biological phenotypes are constrained by finite resources [19, 20]. The evolution of phage resistance may entail fitness costs in host bacteria, limiting indefinite increases in resistance and infectivity, and ultimately resulting in co-existence [8, 14, 21]. In contrast, it is believed that co-evolutionary dynamics in the absence of adaptive cost yield rapid phage extinction [13, 22]. This raises another question: What phage resistance mechanisms lead to trade-offs and further contribute to the different co-evolution outcomes?

Phage resistance, induced by various bacterial extra/intracellular mechanisms, represents a vital phenotype in ecological niches [23–25]. To initiate infection, phages must first attach to specific receptors on the bacterial cell surface [26, 27]. Host bacteria have developed extracellular strategies, such as receptor blocking, competitive inhibitor production, and extracellular matrix production, to prevent the attachment/adsorption process [23, 28]. Upon attachment, phages inject their genetic materials into the host cells to hijack the bacterial machinery, replicate viral nucleic acids and proteins, and assemble and package progeny particles [29]. To combat this, bacteria have developed various intracellular defenses, including the clustered regularly interspaced short palindromic repeat (CRISPR)-CRISPR-associated protein (Cas) system, bacteriophage exclusion (BREX), and abortive infection (Abi) [24, 30–32]. Although many extracellular and intracellular mechanisms have been discovered, the extent to which these mechanisms drive microbial population evolution and ecology remains unclear. For instance, the impact of CRISPR-Cas systems on the ecology and evolution of natural microbial populations was questioned [33]. There are very few wild-type bacteria or archaea for which spacer acquisition from phages or plasmids has been demonstrated to occur at observable frequencies [34, 35]. Moreover, a clear trade-off between CRISPR-Cas systems and horizontal gene transfer has been highlighted, which limits bacterial genome evolution [36]. These findings contradict the hypothesis that CRISPR-Cas systems commonly play a significant role in protecting microbial populations from environmental stresses, such as phages.

In a preliminary study, we observed host-phage co-existence and phage extinction in a 30-day co-evolution experiment using *Salmonella* Enteritidis strains and a lytic phage SF1, which was isolated from our previous research [37]. These interactions provide working models to examine the intrinsic bacterial factors that contribute to phage resistance development and different co-evolutionary outcomes. We hypothesized that host-phage interactions confer new phenotypes to evolved bacterial populations (e.g., phage resistance) and viral populations (e.g., host specificity). Certain bacterial extracellular and intracellular mechanisms, individually or cooperatively contribute to the development of phage resistance, and the trade-offs between developed resistance and fitness could lead to distinct evolutionary outcomes. Additionally, we aimed to uncover the molecular basis of developed phage resistance to better understand the co-evolutionary dynamics.

## Materials and methods

### Strains and preliminary experiment of resistance development

Three *Salmonella enterica* serotype Enteritidis strains, S3 (isolated from Human), S187 (leafy greens), and S5–483 (Human), were collected from the *Salmonella* Foodborne Syst-Omics database [38]. Phage SF1 was isolated from cattle feces collected in Greater Vancouver, British Columbia, Canada, using strain S5–483 as a host. SF1 can form clear plaques on the lawns of three *Salmonella* strains (data not shown), indicating its good infectivity. Lysogeny analysis of SF1 with three selected *Salmonella* strains was conducted [37, 39]. Ten-fold serial dilutions of phage lysates (~10^10^ PFU/ml) were spotted onto lawns of *Salmonella* strains. Resistant cells from the spot centers were re-streaked five times on tryptic soy agar (TSA) to lower phage carry-over. Twenty colonies were randomly collected from the fifth streak. After patch screening, colonies showing plaques or halos were further purified by streaking twice on TSA plates to further minimize phage carry-over [40]. Following the purification, the potential lysogens were suspended in tryptic soy broth (TSB), and cultured at 37°C for 20 h (200 rpm). Each culture was tested for spontaneous phage release by spotting 5 μl of supernatant onto a lawn of host cells. The twenty resistant colonies were further induced via the SOS response using mitomycin C. Overnight cultures were treated with mitomycin C (final concentration: 0.5 μg/ml) and incubated at 37°C for 16 h. The supernatants were filtered (0.45 μm) and spotted onto host overlays [39]. Plaques were visualized after overnight incubation at 37°C. Three biological replicates were conducted.

Bacterial suppression experiments were conducted to determine the emergence of resistance [41]. In a 96-well plate, 100 μl *Salmonella* cells (2 × 10^5^ CFU/ml) in TSB were well mixed with 2 × 10^7^ PFU/ml SF1 particles (multiplicity of infection, MOI 100). The plate was incubated at 37°C for 16 h in a plate reader (Molecular Devices) and optical density at 600 nm (OD_600_) was measured every 30 min. The potential bacterial-resistant community (RC) was collected after 12 h (early stationary phase), washed three times to remove most of the phage particles, and then diluted to around 2 × 10^5^ CFU/ml. The suppression effect of SF1 against the potential RC at MOI 100 was further conducted by mixing 100 μl diluted RC cells with 100 μl 2 × 10^7^ PFU/ml SF1 particles (MOI 100), and culturing at 37°C for 16 h. The measured growth curves (OD_600_) were analyzed by Baranyi and Roberts model using DMFit (https://browser.combase.cc/DMFit.aspx). Lag phage duration (h) was used to estimate the suppression effect of phage SF1 against *Salmonella* cells. Phage densities in the suppression test were determined using the following method. Mixed cultures were collected at intervals, treated with 5% (v/v) chloroform, and centrifuged (13 000 × *g*, 1 min). The lysates were then diluted in SM buffer, and 5 μl aliquots were spotted on infused soft agar (TSB with 0.7% w/v agar and inoculated with 10^8^ CFU/ml host cells) for enumeration [42].

### Co-evolution experiment

**Figure 1:**
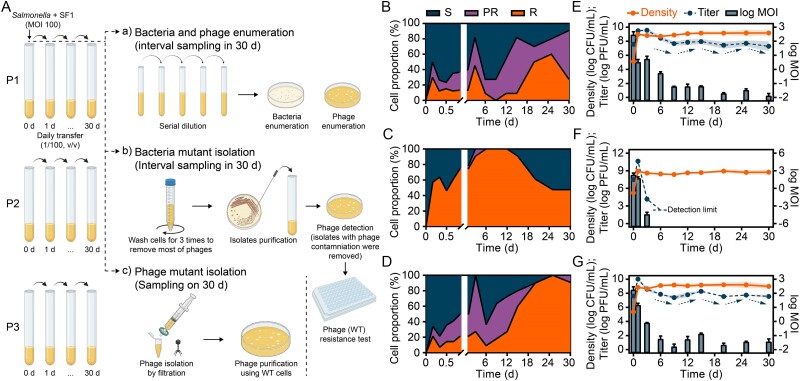
**Co-evolution of *Salmonella* Enteritidis strains with phage SF1**. **A**) a schematic diagram represented a 30-day co-evolution test. Three *Salmonella* strains (three replicates per strain) were propagated with the lytic phage SF1 and continuously transferred for 30 days. Figure created with BioRender.com. **B**–**D**), proportion changes of sensitive (S), partial resistant (PR), and completely resistant (R) populations in the evolutionary groups of S3 (**B**), S187 (**C**), and S5–483 (**D**). For each group at each timepoint, bacteria-phage mixtures were collected and washed three times to remove most of the phage particles. They were then spread on plates and 45 evolved isolates were purified. Their phage resistance patterns were examined using 96-well plates and a plate reader, using a liquid medium-based assay. **E**–**G**) changes in total bacterial density, phage titer, and log MOI in S3 (**E**), S187 (**F**), and S5–483 (**G**) evolution groups. Before daily transferring, bacteria-phage mixtures were sampled, and bacteria enumeration was performed. After filtration, phage titer in the medium was measured on infused soft agar. Bacteria density and phage titer are shown by solid and dashed lines, respectively, with the shaded area indicating mean ± s.d. (n = 3). Dashed lines with arrows indicate phage titer dynamics. Log MOI was calculated based on bacteria density and phage titer measurements at each time point and represented by bar graphs. Error bars represent one s.d. above the mean values (n = 3).

To set up the co-evolution experiment, three colonies of each *Salmonella* strain were picked from plates and cultured in TSB overnight (Fig. 1A). Subsequently, they were diluted to 10^5^ CFU/ml in glass tubes and inoculated with 10^7^ PFU/ml SF1 particles. The tubes were incubated at 37°C with shaking at 200 rpm. After 24 h, 100 μl of each community was transferred into a new tube containing 10 ml fresh TSB. This daily transfer process was conducted for a duration of 30 days. Interval sampling was performed to determine bacteria and phage densities, as well as to preserve mixture communities for later analysis. For bacteria enumeration, aliquots were diluted in peptone water, and plated on TSA. For phage quantification, aliquots were filtered (0.45 μm), diluted in SM buffer, and spotted (5 μl) on infused soft agar mentioned above.

To isolate evolved bacterial strains, preserved mixture communities were washed three times to remove phage particles (Fig. 1A). Washed cells were streaked on TSA and incubated overnight at 37°C. Then, a total of 15 colonies were isolated from each plate (15 × 3 for each *Salmonella* strain), and overnight cultured in TSB. Phage contamination in cultures was tested on a double layer, and isolated strains cleaned of phage were preserved. To isolate evolved phages on Day 30, preserved communities were filtered (0.45 μm), diluted in SM buffer, and mixed with TSB soft agars (55°C) inoculated with 10^8^ CFU ancestral host cells. The mixed soft agars were poured over TSA plates and incubated overnight at 37°C. Plaques were isolated from these plates and further purified three more times. Then, purified phages were propagated in ancestral host cells [42]. Ancestral host cells were used to screen and propagate evolved phages, as those capable of infecting ancestral host cells predominated in the evolved phage population. The highest phage titers were recorded when using ancestral host cells compared to evolved strains (e.g., S5–483R2 and S5–483R3) as hosts (data not shown). In another co-evolution test, SF1 particles were daily added (final concentration: 10^9^ PFU/ml) to keep MOI at 100 during transfer. Tests including enumeration of bacteria and phages, and isolation of evolved bacterial strains, were conducted as the same as mentioned above (Fig. S1a).

### Emergence of resistance

To investigate the patterns of developed phage resistance, we assessed a total of 135 evolved isolates (15 evolved isolates × 3 replicative population × 3 *Salmonella* strains) at each sampling point. The resistance assays were conducted in liquid medium using 96-well plates. For each isolate, an overnight culture in TSB was diluted to 10^5^ CFU/ml. Subsequently, treatment groups were inoculated with phage SF1 (final titer: 10^7^ PFU/ml). The OD_600_ of these mixtures was continuously monitored by plate reading over a 16 h incubation period at 37°C. By comparing the growth dynamics of ancestor strains under phage treatment, we were able to classify the evolved isolates into different resistance patterns, including sensitivity (S), partial resistance (PR), and complete resistance (R). More specifically, growth curves were analyzed using the Baranyi and Roberts growth model with DMFit (https://browser.combase.cc/DMFit.aspx) to obtain the lag phase duration (Table S1). The lag phase extension (lag phase duration of the phage-treated group minus the lag phase duration of the control group) of the evolved strain was compared to that of the ancestral group. An evolved strain was defined as S if the lag phase extension of the evolved strain was not significantly different from that of the ancestral group, PR if the lag phase extension of the evolved strain was significantly lower than that of the ancestral group, and R if the lag phase extension of the evolved strain was ~0 (lag phase extensions of the phage-treated group and control group were not significantly different). To further create an indicative scale of resistance, we input the OD_600_ trajectories of bacterial cultures exposed to phage treatments into principal component analysis (PCA) [15, 43]. The PCA results showed that principal component 1 (PC1) explained at least 81.3% of the variance and effectively differentiated between S, PR, and R patterns along a continuous scale.

### Phage adsorption rate and relationship with resistance

To determine whether the evolved phage resistance resulted from blocked cell surface attachment, we carried out an adsorption assay [44]. Representative S, PR, and R isolates were selected at the latest timepoint at which resistance was detected. Phage SF1 was added to exponentially growing evolved isolates (10^8^ CFU/ml) at a MOI of 5. Sampling was performed at intervals from the start of phage addition to 2 h of incubation. The collected samples were immediately filtered to remove cells, and phage titers in the extracellular medium were then measured by the spot test on infused soft agar. A decline in phage titers in the filtered samples over a specific time period (maximum adsorption) indicates attachment. Average adsorption rates were calculated based on the dynamics of titer decline. The period of maximum adsorption ranged from 20 to 40 min, depending on the bacterial strains. Linear model tests were then conducted to examine the relationship between PC1 values mentioned above (used as resistance scores) and calculated average adsorption rates.

### Phage fitness cost assay

Growth competitions were conducted between PR and S isolates (PR + S), as well as R and S isolates (R + S), to measure the fitness cost of phage resistance development [15]. Colonies of evolved isolates were picked and cultured overnight. The cultures were then diluted to around 10^5^ CFU/ml in TSB, and aliquots were diluted and plated on TSA plates to determine the exact initial densities (T_0_) of each isolate. The diluted cultures (around 10^5^ CFU/ml) of paired isolates (PR + S or R + S) were mixed at a 1:1 ratio (v/v) and incubated at 37°C for 24 h. After that, aliquots were diluted and plated on TSA to determine the final densities (T_F_) of the PR and S mixture or R and S mixture. To distinguish between the PR (T_F-PR_) and S density (T_F-S_) or R (T_F-R_) and S density (T_F-S_) within the mixed T_F_, 20 colonies were randomly collected from TSA measuring T_F_, cultured overnight, and diluted to 10^5^ CFU/ml. Their resistance patterns were further determined in 96-well plates, as mentioned above in the “Emergence of resistance” section, to identify the colonies of S (C_S_), PR (C_PR_), and R (C_R_). Therefore, T_F-S_ = (C_S_/20) × T_F_, T_F-PR_ = (C_PR_/20) × T_F_, and T_F-R_ = (C_R_/20) × T_F_. Lastly, the relative fitness (*W*) of evolved PR and R strains was calculated using the formula *W* = M_A_/M_B_, with M_A_ = ln (T_F-PR_/T_0_) for PR isolates, M_A_ = ln (T_F-R_/T_0_) for R isolates, and M_B_ = ln (T_F-S_/T_0_) for S isolates.

### Isolation and characterization of evolved phages

After 30 days of co-existence with *Salmonella* S3 or S5–483, we isolated, purified, and characterized evolved phages with different plaque morphologies (e.g., size). To determine their host ranges against S, PR, and R bacterial isolates, phages were propagated with wild-type (WT) cells, and then standardized to a concentration of 10^8^ PFU/ml in SM buffer. Then, 5 μl phage solutions, in duplicate, were spotted onto lawns of evolved isolate cells and incubated at 37°C for 16 h. We evaluated zones of cell lysis using a scaling system, where a score of 0 indicated a zone with complete turbidity (indicating no infection), and a score of +4 indicated a completely clear zone with no turbidity [45]. Suppression abilities of evolved phages against WT cells were further determined in 96 well plates as described in “Strains and preliminary experiment of resistance development”.

### Infection assay

The phage isolate (SF1-S3–1-2) with the best inhibitive ability was chosen, and the following experiments were conducted to characterize its infection properties: an adsorption assay was carried out to determine the surface attachment efficacy (as mentioned in the “Phage adsorption rate and relationship with resistance”); a one-step growth curve was constructed to determine the phage burst size [46]; a real-time RT-PCR (qPCR) was performed to investigate the expression patterns of phage genes during infection. To determine the burst size, overnight cultured S3 cells were diluted to 10^9^ CUF/ml, corresponding to an OD_600_ of 1.0. Phage SF1-S3–1-2 was added at MOI 0.1 and allowed to adsorb for 10 min at room temperature. Excess phage particles were removed by centrifugation at 6000 × *g* for 5 min at 4°C. The pellet was suspended in TSB and incubated at room temperature. Aliquots were collected over a 120 min period, immediately filtered, and spotted onto infused soft agar with WT cells for titer determination.

To investigate phage gene transcription during the latent period, S3 cells collected at the late-log phase (around 10^8^ CFU/ml) were infected with SF1 (control) and SF1-S3–1-2, respectively, at MOI 5. Samples were collected every 5 min until reaching the maximum adsorption point (20 min). These conditions, including using exponential cells, a higher MOI, and sampling within the first-round latent period, ensure a synchronized infection for the subsequent qPCR test. The collected samples were immediately stabilized using RNAProtect bacterial reagent (Qiagen), and total RNA was extracted using a Total RNA Miniprep Kit (New England Biolabs, NEB). RNA quality was assessed using a bioanalyzer (Agilent Technologies). Subsequently, cDNA was synthesized from 1 μg RNA using a LunaScript RT SuperMix Kit (NEB) [47]. The expressions of six phage genes, encoding the following proteins: tail tape measure protein (QDH45043), portal protein (QDH45085), DNA polymerase (QDH45054), DNA helicase (QDH45061), tail fibers protein (QDH45047), and capsid protein (QDH45093), were quantified using Luna Universal qPCR Master Mix (NEB) on a BioRad CFX96 real-time thermal cycler [48]. To quantify the selected genes in mRNA, standard curves were generated from SF1 genomic DNA, which was extracted using a phenol/chloroform-based method. DNA concentrations were measured by Nanodrop (Thermo Fisher Scientific) and converted to genomics copies by entering the phage genome length (41 396 bp) into the URI Genomics & Sequencing Center calculator (http://cels.uri.edu/gsc/cndna.html). The primers used in this study are listed in the Table S2. Three biological replicates were conducted for the qPCR test.

### Efficiency of plating of evolved phages against resistant bacteria

Based on the host range test, evolved phages (SF1-S3–1-2, SF1-S3–1-4, and SF1-S3–2-1) which exhibited strong lytic abilities against the completely resistant strain S3R3, were selected for determining their efficiency of plating (EOP). These phages were propagated using ancestor S3 cells and standardized to a concentration of 10^8^ PFU/ml in SM buffer. Tenfold dilutions of the phage solutions were spotted (5 μl) onto infused soft agar plates with S3 or S3R3 cells. The EOP on the resistant host was calculated using the formula: EOP = (titer on S3R3) / (titer on S3) [49]. To check if evolved phages were able to replicate during infection, a suppression test of selected evolved phages against S3R3 was further conducted. S3R3 cells (10^5^ CFU/ml) were mixed with each evolved phage (10^7^ PFU/ml) and incubated at 37°C for 16 h. Interval sampling was performed, and bacterial and phage densities were enumerated.

### Whole genome sequencing of evolved bacterial and phage mutants

Bacterial colonies were overnight cultured in Luria-Bertani (LB) broth at 37°C with agitation at 200 rpm. Bacterial genomic DNA was extracted from the cultures using a DNeasy Blood & Tissue Kit (Qiagen). Phage DNA was extracted using a phenol/chloroform-based method. DNA concentrations were quantified using a Qubit Fluorometer 3.0. Subsequently, DNA libraries were constructed using the DNA Prep workflow (Illumina) and multiplexed for sequencing runs to achieve a depth of coverage exceeding 25×. Paired-end whole genome sequencing (WGS) was performed on a MiSeq instrument (Illumina) using V2 chemistry, resulting in fragment lengths of 2 × 150 base pairs (300 cycles) [50].

### Genome assembly and analyses

To assemble the genomes of the WT bacterial strains, the pair-end reads (FASTQ) were subject to quality control using FastQC (v.0.12.1), then processed by Trimmomatic (v.0.38) to remove adapter sequences and low-quality bases. Another round of FastQC was performed to confirm the improved read quality after trimming. The filtered reads were subsequently assembled using Unicycler (v.0.5.0), and the quality of the assemblies was assessed using QUAST (v.5.2.0). The obtained contigs were further rearranged and ordered using the multi-reference scaffolder MeDuSa (v.1.6), with the reference genome obtained from KmerFinder (v.1.2) [51]. To reduce assembly gaps for scaffolding, Soapdenovo2 Gapcloser (v.1.12) was employed. Finally, the bacterial genomes were annotated using Prokka (v.1.14.6). To identify nucleotide mutations in evolved bacterial and phage mutants, their raw reads were compared to WT genomes using Breseq (v.0.35.5), which is a computational pipeline designed for analyzing short-read resequencing data. We applied the default Breseq parameters, which included using consensus mode with a consensus frequency cutoff of 0.8 and a consensus mutation E-value cutoff of 10 [52]. Based on bacterial mutation profiling, gene ontology-enrichment analysis (http://geneontology.org/) was performed to investigate whether mutations were associated with any specific gene ontology or pathway term [53].

### Mutation rate of *Salmonella* strains under phage stress

The resistant mutation rates (μ) of three *Salmonella* strains under SF1 treatment were determined using the Luria–Delbrück fluctuation test [15]. Three WT strains were cultured overnight in TSB, then diluted and inoculated into independent isogenic cultures (10^3^ CFU/ml) in a 96-well plate. These cultures were allowed to grow overnight. Subsequently, the cultures were further diluted, and 10 μl of each culture was inoculated into a new plate containing 150 μl TSB and 50 μl phage solution (final concentration: 10^9^ PFU/ml) in each well. Fluctuation tests were conducted with 10^4^–10^5^ cell inoculums. The plates were incubated at 37°C and monitored for 24 h. Wells with no observable growth were deemed cells without mutations. To calculate the mutation rate μ, the P_0_ method (μ = −ln(P_0_)/N) was applied, where P_0_ represents the fraction of cultures without resistant mutations, and N is the population size.

### Mutant construction and characterization

Based on the WGS profiling data, genes that exhibited mutations during co-evolution were selected and deleted to study their functions in the development of phage resistance. To achieve this, we generated allelic exchange plasmids by cloning ~0.8 kb homology arms that flanked the genomic region targeted for deletion into the suicide vector pRE112 [54]. These plasmids were then transformed into SM10λpir cells using the CaCl_2_ method [55]. Following extraction, they were transformed into WT S5–483 cells by electroporation (1.8 kV, 2.5 ms) to induce a first crossover event on selective agar. The single-crossover strains were cultivated in LB containing 8% (w/v) sucrose to facilitate a second crossover. Colonies that displayed resistance to sucrose but S to chloramphenicol were carefully selected [56]. The resulting mutants were confirmed through PCR analysis and DNA sequencing. To generate complemented strains, deleted genes with homology arms were cloned from WT cells and introduced into pRE112. Constructed plasmids were then transferred into the corresponding mutant strains by electroporation at 1.8 kV for 2.5 ms. A double selection was then carried out as described above. The complementation of these genes was confirmed by PCR and DNA sequencing.

To gain insights into the functions of the deleted genes in the development of phage resistance, we carried out a series of experiments: the EOP of SF1 against the mutants, as described in “Efficiency of plating of evolved phages against resistant bacteria”, and phage suppression effect on the mutants and complemented cells, as described in “Strains and preliminary experiment of resistance development”, were tested; adsorption assay was conducted on selected mutants to assess the phage adsorption rate; qPCR test was performed to confirm the *rfaH*-reguated *rfa* and *rfb* cluster genes in the cells of RC and Δ*rfaH* at the early stationary stage. Lastly, to validate the role of the key gene *rfaH* in developing complete resistance, we deleted the *rfaH* gene, which contained a nonsense mutation in the evolved mutant S5–483R1, using the method described earlier. Subsequently, we introduced a normal *rfaH* gene, amplified from WT cell, into S5–483R1 after deleting its native *rfaH* gene. Spot and suppression tests were performed to assess changes in phage-resistant phenotypes resulting from the genetic modification.

## Results and discussion

### Co-existence and phage extinction in host-phage co-evolution

In this study, three *Salmonella* Enteritidis strains (S3, S187, and S5–483) were chosen because they are susceptible to infection by phage SF1, as demonstrated in a host range test from our previous work [37]. SF1 is a strictly lytic phage for the three strains, as evidenced by the absence of released phage particles in resistant colonies during the lysogeny analysis. In a preliminary test, these three strains exhibited comparable lag phase durations (4.0–5.4 h) when adapting to SF1 phage stress (MOI 100) after co-cultivation for 12 h (Table S3). Additionally, the recovered population of these strains indicated the successful development of phage resistance (Fig. S2a-c). An MOI of 100 was used to simulate phage therapy, as MOI values greater than 100 are recommended for *ex vivo* and *in vivo* applications [6].

To confirm the developed resistance, the RC at 12 h was collected, washed, diluted, and then re-treated by phage at MOI 100. The S187 RC developed stronger resistance compared to the other two groups, as indicated by its shorter lag phase extension (0.3 h) compared to S3 RC (1.0 h) and S5–483 RC (1.7 h) groups under repeated phage stress (Table S3). Moreover, S187 RC efficiently suppressed the synthesis of SF1 during infection, whereas the other two groups could not (Fig. S2d-f). Based on the data collected (confirmation of resistance development and appropriate initial MOI) in a preliminary test, long-term co-evolution studies were set up. Three *Salmonella* strains were mixed with the lytic phage SF1 at MOI 100 on the first day and then transferred daily at a ratio of 1/100 (v/v) for 30 days (Fig. 1A). In another supplementary study, in addition to the initial mixture of *Salmonella* and phage, SF1 was added daily to the mixture during the 30 days’ transfers to maintain an initial MOI 100 (Fig. S1a).

Three distinct resistant patterns, including S, PR, and R, were consequently documented in evolved cells by statistically comparing their lag phase extensions with those in ancestral host cells (Fig. S3, Fig. S4, Fig. S5 and Table S1). An isolate was classified as S if it exhibited the same cell density dynamics as WT cells in the presence of phages, and R if it showed uninhibited bacterial growth. The PR pattern was characterized by cells displaying less growth inhibition compared to S cells [15, 57]. In the study with daily phage complementation, R isolates dominated the bacterial population of S3, S187, and S5–483 groups after co-evolving with SF1 for 12, 3, and 24 days, respectively (Fig. S1). S and PR cells were nearly eliminated under successive phage challenges. Among the three strains, S187 developed the fastest strong resistance, reaching a 100% R population by day 3, compared to the other two strains. This conclusion was consistent with the preliminary experimental results (Fig. S2). In contrast, the co-evolution test without the phage complementation displayed fluctuating selection dynamics. Two distinct evolutionary outcomes, co-existence or phage extinction, emerged within the three groups (Fig. 1B-D).

S187 effectively defended against SF1, eradicating it from the co-evolutionary system after a 6-day co-evolution (Fig. 1F). Moreover, during co-evolution, R populations appeared to be favored over PR populations: PR frequency was much lower, with only one isolate obtained. Subsequently, from Day 12, the proportion of S population slowly recovered to 52.4% at the end (Day 30), suggesting that some intrinsic factors further shaped the bacterial community (Fig. 1C). Meanwhile, S3 and S5–483 co-existed with SF1 throughout the 30-day test. PR and R populations showed fluctuating increases (Fig. 1B, D), whereas the phage population experienced fluctuating decreases (Fig. 1E, G). Phage extinction is a frequent occurrence in host-phage co-evolution [58]. Nevertheless, some studies have demonstrated long-term arms races, indicating no fundamental constraint on phages’ co-evolutionary ability [8, 13, 24, 59]. In our case, strain S187 successfully eliminated the parasite (i.e. SF1) after 6 days of adaptation, whereas strains S3 and S5–483 co-existed with the phage throughout 30 days of co-evolution.

### Phage resistance is determined by phage adsorption rate

**Figure 2:**
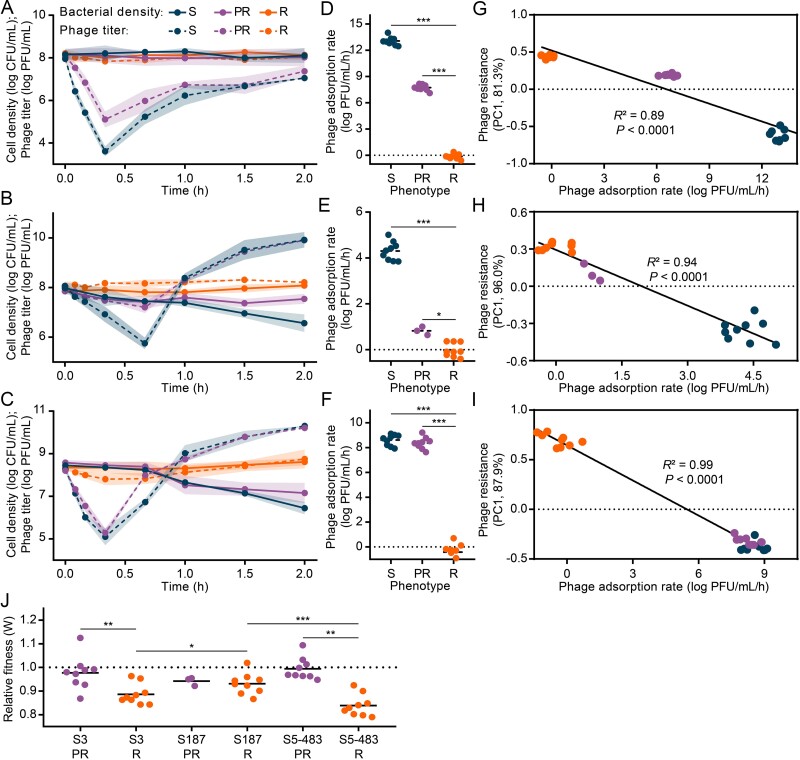
**Development of phage resistance and related fitness cost**. **A**–**C**), attachment dynamics of SF1 onto isolates evolved from S3 (**A**), S187 (**B**), and S5–483 (**C**) (S, sensitive; PR, partial resistant; R, completely resistant). These isolates were mixed with SF1 at MOI 5, and the mixtures were sampled at intervals. Samples were immediately filtered, and the phage titers were then determined. Bacteria density and phage titer during attachment are shown by solid and dashed lines, respectively, with the shaded area indicating mean ± s.d. (n = 9, 3 isolates × 3 replicates). **D**-**F**) average phage adsorption rates of isolates evolved from S3 (**D**), S187 (**E**), and S5–483 (**F**). Rate values were calculated from the adsorption phases (first 20 min for S3 and S5–483 groups, and 40 min for S187 group, see **A**-**C**). The asterisks above dots indicate significant pairwise differences using *t* test (^*^P < 0.05, ^*^^*^^*^P < 0.001). **G**-**I**) the linear correlation between phage resistance (described by PC1, Fig. S7a-c) and adsorption rate (retrieved from **D**-**F** and Fig. S6a-c) of isolates evolved from S3 (**G**), S187 (**H**), and S5–483 (**I**). Isolates were color-coded as in **A**. *R*^2^ showed fit goodness, and statistical significance was calculated by a two-tailed F test. **J**) fitness of evolved PR and R isolates relative to S isolates (n = 9, 3 isolates × 3 replicates; for S187 PR, n = 3, 1 isolate × 3 replicates). The asterisks indicate significant pairwise differences using one-way ANOVA, post hoc LSD test (^*^P < 0.05, ^*^^*^P < 0.01, ^*^^*^^*^P < 0.001).

It is essential to determine the specific resistance mechanisms that evolved during co-evolution. The extracellular mechanism is manifested by the lack of attachment to the cell surface, and intracellular resistance is associated with various mechanisms, such as the CRISPR-Cas system [24, 60, 61]. In this study, we first assessed phage adsorption, which is a key aspect of the extracellular mechanism [62, 63]. Cells were mixed with SF1 at MOI 5, and the phage titers in the medium of S and PR isolates continuously declined (Fig. 2A-C). For R isolates, phage attachment was effectively blocked, maintaining stable phage titers in the medium. After incubation for 20 min, phage bursts were observed in S3 and S5–483, while the burst time was delayed to 40 min in S187 group. The decline in phage titer during the adsorption process showed a strong linear correlation across the three groups (Fig. S6). Therefore, the average adsorption rates (slope values) before the burst of evolved isolates were calculated from linear fitting during the adsorption phase (first 20–40 min) (Fig. 2D-F). S3 S isolates exhibited the highest adsorption rate at 13.1 log PFU/ml/h, followed by S isolates of S5–483 (8.6 log PFU/ml/h) and S187 (4.3 log PFU/ml/h). The lower adsorption rate in S187 could potentially make it less susceptible to SF1.

The correlation between phage resistance and adsorption rate was further examined. To mathematically describe the resistant patterns, PCA, which was able to transform and score growth trajectories, was employed (Fig. S7). PC1, which described the majority (> 81.3%) of the data, successfully discriminated different resistance levels along a continuous scale. Intriguingly, strong linear correlations (*R*^2^ > 0.89, linear regression analysis, t-test, *P* < 0.0001) were observed between phage resistance and adsorption rate in all three groups (Fig. 2G-I). These results confirmed that certain extracellular mechanisms, such as receptor blocking, outer membrane vesicle, or exopolysaccharides, significantly contribute to *Salmonella*'s phage resistance by preventing the adsorption of SF1. The exact mechanism will be explored further in this study. Moreover, such passive extracellular defenses are believed to be more efficient for the host compared to intracellular mechanisms, as the phage does not even enter the cell [44].

### Fitness cost of phage resistance development

Surface modification usually impacts specific membrane functions, such as membrane integrity and motility, resulting in fitness costs [8, 64]. Therefore, we conducted competition experiments in which PR or R isolates were paired with S cells, to determine the fitness cost. It turned out that developing R phenotype was more costly than PR for cells evolved from S3 and S5–483 (Fig. 2J). Only small costs (relative fitness, *W*) were recorded in the S3 PR ($\overline{W}$ = 0.98) and S483 PR ($\overline{W}$= 0.99) populations. Moreover, the relative fitness of S3 R *(*$\overline{W}$ = 0.89, one-way analysis of variance (ANOVA), post hoc LSD test, *P* = 0.047) and S5–483 R ($\overline{W}$= 0.84, one-way ANOVA, post hoc LSD test, *P* < 0.05) isolates was significantly lower than that of S187 R ($\overline{W}$= 0.93). Thus, the higher fitness costs in S3 R and S5–483 R populations probably prevented host resistance from increasing indefinitely, resulting in co-existence (Fig. 1B, D). Additionally, the relative cost between PR ($\overline{W}$= 0.94) and R ($\overline{W}$= 0.93) populations in the S187 group was not significant (one-way ANOVA, post hoc LSD test, *P* = 0.75) (Fig. 2J). This explains the low frequency and eventual loss of the S187 PR population during co-evolution because the low cost of the S187 R population was favored under phage stress. Furthermore, the survived S population slowly recovered after phage removal due to the cost of R cells (Fig. 1C).

### Co-evolved phages contribute to bacterial evolutionary dynamics

**Figure 3:**
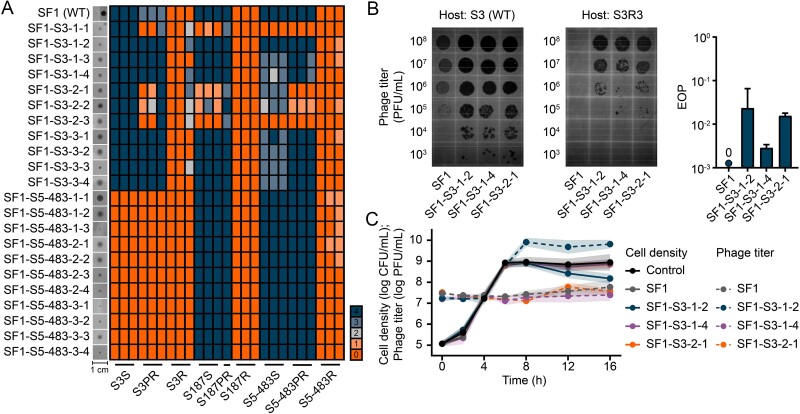
**Evolved phages with enhanced infectivity abilities**. **A**) images on the left of the heatmap shows the plaque morphologies of evolved phages, which were labeled as ancestor phage-evolutionary host-replicative population-isolate number. For ancestor phage SF1, its plaques on S3 (left) and S5–483 (right) lawns are presented. The heatmap shows the qualitative level of clearing (0–4; 0, no lysis; 4, full clearance without turbidity), produced by evolved phages on soft lawns of evolved bacterial isolates. **B**) the efficiency of plating (EOP) of evolved phages when infecting both the wild-type (WT) S3 and the evolved R strain S3R3. Evolved isolates were labeled as ancestor strain, evolved resistant pattern and isolate number. Left, serial, tenfold dilutions of the indicated phages spotted on lawns of S3 and S3R3. Right, EOP data were calculated accordingly. **C**) suppression effects of evolved phages against strain S3R3. Bacteria density and phage titer during co-culture are represented by solid and dashed lines, respectively, with shaded area indicating mean ± s.d. (n = 3).

Although the co-evolution mechanisms remain elusive, there is no doubt that phage evolution plays an important role in shaping bacterial populations [65, 66]. To better understand the bacteria-phage interaction, the evolved phages were isolated, purified, and characterized. A total of 22 phages co-existed with S3 or S5–483 were obtained. They exhibited various plaque morphologies on lawns of ancestor hosts, indicating potential altered genetic information (Fig. 3A). Moreover, the host range and growth suppression tests showed that the evolved phages became more specific to the bacteria they evolved with. For instance, phages co-evolved with S5–483 were no longer able to infect S3 populations (Fig. 3A). These altered phenotypes resulted from an arms race between host and parasite. The host bacteria in this study evolved phage resistance by blocking adsorption, thus, phages must evolve to recognize the modified receptors. Because bacterial surface modification strategies can be strain-dependent, phage evolution may lead to increased specificity [67].

The arms races also conferred favorable phenotypes on evolved phages [68, 69]. For instance, SF1-S3–1-2 gained a better inhibitive effect against ancestor S3 cells (Fig. S8a and Table S4). It prolonged the lag phase extension of the bacteria-phage mixture for 20.4 h, which was significantly (one-way ANOVA, post hoc LSD test, *P* < 0.0001) longer than that of the ancestor SF1-treated group (5.5 h). The following adsorption and one-step growth curve tests demonstrated that the burst size of SF1-S3–1-2 was about four times higher than that of SF1 (Fig. S8b, c). We examined the expression patterns of six selected phage genes related to accessory and structural functions, as well as DNA metabolism (Fig. S8d). It showed that at the later stage (15–20 min) before burst, phages genes, which coded portal protein (QDH45085) and capsid protein (QDH45093), were significantly (one-way ANOVA, post hoc LSD test, *P* < 0.001) induced in SF1-S3–1-2 treated group (Fig. S8e). The mutation of certain regulator genes may activate phage gene transcription [70, 71]. The increased burst size may lead to higher MOI and enhance the inhibitory effect of evolved phages.

Some evolved phages (e.g., SF1-S3–1-2 and SF1-S5–483–1-1) were able to infect some R bacterial isolates (e.g., S3R3, S5–483R2, and S5-483R3), which blocked the adsorption of ancestor phage (Fig. 3A). In contrast, R isolates evolved from S187 developed unbeatable resistance, with neither WT nor evolved phages able to infect them. Three evolved phages (SF1-S3–1-2, SF1-S3–1-4, and SF-S3–2-1) with good lytic abilities (lytic score 3) against S3R3, were selected for further tests. The EOP test showed that their infectivity was 42.9–350.0 times lower than that of the ancestor phage SF1 (Fig. 3B). Suppression assays showed that the evolved phage SF1-S3–1-2 reduced host cell density and propagated to ~10 log PFU/ml. Whereas the other two isolates did not exhibit inhibitory effects, and their propagations were also limited (Fig. 3C). Because the inoculation MOI 100 was calculated using WT cells, the exact MOI could be significantly lowered, considering the decreased infectivity. A low MOI might limit the lysis of infected cells, and phages could not replicate sufficiently [72]. Collectively, the arms race conferred various altered phenotypes on phages. Some evolved phages acquired the ability to infect and propagate within certain R cells, which incurred fitness costs, in co-existence groups. The costly and defeatable R phenotype made it more difficult for the host bacteria to eliminate the parasites, resulting in co-existence.

### Genomic profiling reveals the arms race during host-phage co-evolution

**Figure 4:**
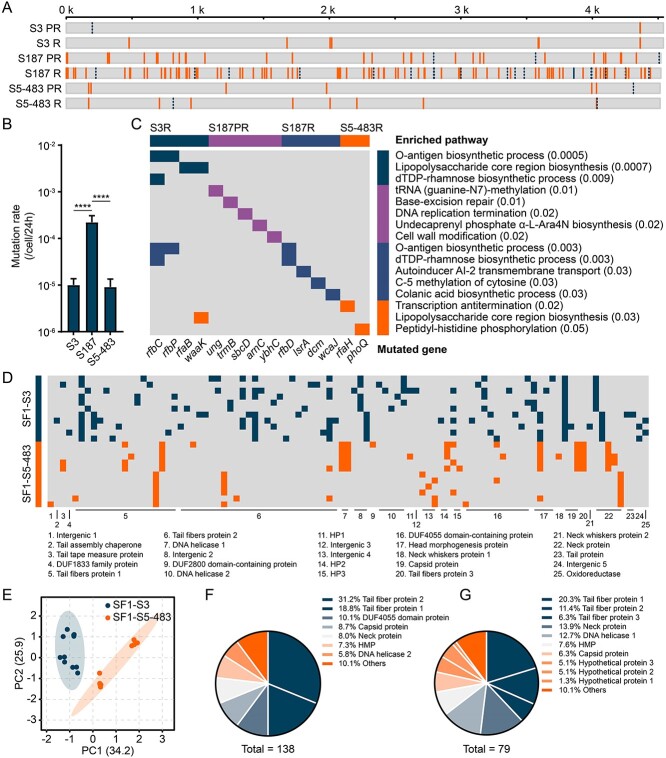
**Genomics profiling of evolved *Salmonella* and phage isolates**. **A**) mutation distribution across the genomes of evolved PR and R isolates. Mutations in coding and intergenic regions are shown with in solid and dash lines, respectively. The scale bar indicates gene numbers in the genome. **B**) mutation rates of three *Salmonella* strains under phage SF1 stress. For each strain, 20 cultures were analyzed, as determined by fluctuation assays. Data are graphed as mean ± s.d. and the asterisks above bars indicate significant pairwise differences using one-way ANOVA, post hoc LSD test (^*^^*^^*^^*^P < 0.0001). **C**) the most significantly enriched mutation gene region in gene ontology biological process categories. For the complete enrichment, see Table S8. **D**) mutation profiling of phages evolved with strain S3 (SF1-S3) and S5–483 (SF1-S5–483). These mutations concentrated in 20 proteins and five intergenic regions. For the detailed mutation profiles in each phage isolate, see Table S9. **E**) principal components analysis of mutation profiles of evolved phages. Each dot represents a phage isolate. Phages evolved with S3 and S5–483 were well separated. Shaded areas indicated 95% confidence. **F**, **G**) the distribution of major mutations in the phages that evolved with S3 (**F**) and S5–483 (**G**). For both groups, mutations were enriched in the tail fiber proteins 1 and 2, indicating their critical role in evolution.

To understand the bacterial resistant phenotype and viral adaptation during co-evolution at the genomic level, we sequenced the whole genomes of representative isolates. From evolved bacterial isolates, various mutation types (e.g., frameshift, point, and nonsense mutations) were recorded (Fig. 4A, Tables S5, S6, and S7). S187 R isolates had the highest number (107) of mutations, followed by S187 PR group (38). Much fewer mutations were found in S3 PR (8), S3 R (11), S5–483 PR (7), and S5–483 R (12) isolates. Compared to S3 and S5–483, strain S187 accumulated more mutations during the co-evolution with phage SF1. We further confirmed the conclusion by measuring the mutation rates of these three strains under phage stress (Fig. 4B). Using P_0_ method, we estimated a resistant mutation rate of 2.2 × 10^−4^/cell/24 h for S187, which was significantly higher than those in S3 (1.0 × 10^−5^/cell/24 h, one-way ANOVA, post hoc LSD test, *P* < 0.0001) and S5–483 (0.9 × 10^−5^/cell/24 h, one-way ANOVA, post hoc LSD test, *P* < 0.0001). Mutations in genes involved in maintaining bacterial genome integrity and DNA repair, such as *dcm*, *ung*, *sbcC*, and *sbcD*, may contribute to more mutations in S187 (Table S6) [73–75]. Moreover, the accumulated mutations in S187 may increase the probability of an adaptive mutation under selection, enabling it to develop faster and stronger resistance, ultimately leading to phage extinction [76, 77].

Further pathway analysis showed no significant pathway in S3 PR and S5–483 PR isolates (Fig. 4C and Table S8). Contrarily, some intracellular biological pathways, such as tRNA methylation, and extracellular pathways (e.g., cell wall modification) were significantly affected by the mutations in S187 PR (Gene Ontology analysis, hypergeometric test, *P* < 0.05). This implies that both mechanisms jointly contribute to PR development, and neither of them alone is sufficient to eliminate phage stress. Additionally, it showed that for S3 R and S187 R isolates, the O-antigen biosynthesis process was the top altered pathway. Moreover, transcription antitermination, which was regulated by gene *rfaH*, was the most influenced pathway in S5–483 R isolates. The gene *rfaH* controls the expression of O-antigen and lipopolysaccharides (LPS) core synthesis related genes [78, 79]. This implies that O-antigen could be the target receptor of phage SF1.

The genetic evolution of phage SF1 during co-evolution was also investigated (Fig. 4D and Table S9). More mutations were recorded in phages evolved with S3 (SF1-S3, n = 138), compared with those evolved with S5–483 (SF1-S5–483, n = 79). In addition, 31.2% and 18.8% mutations concentrated in tail fiber proteins 2 and 1, respectively, in SF1-S3 phages. While in SF1-S5–483 phages, nucleotide alterations in fiber proteins 1, 2, and 3, were accounted for 20.3%, 11.4%, and 6.3% of the mutations (Fig. 4E-G). It has been well documented that phage tail fiber protein plays a key role in recognizing receptors and initiating infections [80, 81]. Collectively, the mutation profiling was in consistent with the conclusion from Fig. 2: surface modification (O-antigen in this study) determined the phage resistance development. To counteract, phages had to evolve their tail fiber proteins to recognize the modified receptor or improve the binding efficacy.

### Gene deletion reveals the critical role of O-antigen defects in co-evolution

**Figure 5:**
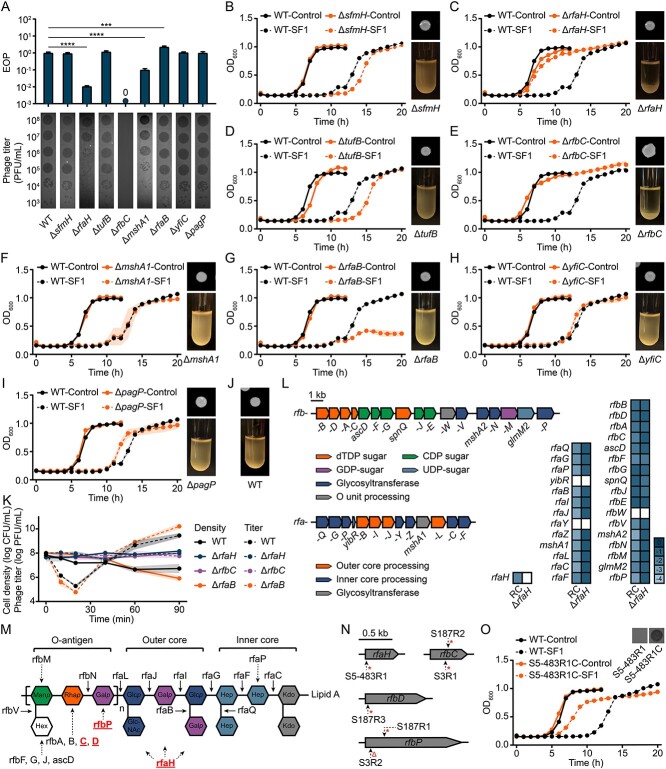
**The critical role of *rfaH*-regulated *rfb* genes in phage resistance development during evolution**. **A**) the efficiency of plating (EOP) of SF1 when infecting wild-type (WT) and constructed mutants. Top, phage resistance by EOP for mutants with deleted genes (n = 3). The asterisks above bars indicate significant pairwise differences using one-way ANOVA, post hoc LSD test (^*^^*^^*^P < 0.001, ^*^^*^^*^^*^P < 0.0001). Bottom, serial, tenfold dilutions of SF1 spotted on lawns of WT and mutants. **B**–**I**) left, suppression test of SF1 against constructed mutants. Lines with shaded area indicate mean ± s.d. (n = 3). Top right, colony morphology of mutants grown on TSA plates. Images were acquired after 16 h of growth at 37°C. Bottom right, flocculation behaviors of mutants in TSB, after letting stand at room temperature for 2 h. **J**) Colony morphology (top) and flocculation (bottom) of WT cells. **K**) attachment dynamics of SF1 onto WT and selected mutants. Bacteria density and phage titer during attachment are shown by solid and dashed lines, respectively, with shaded area indicating mean ± s.d. (n = 3). **L**) left, *rfa* and *rfb* clusters in the S5–483 genome. Right, heatmap of relative expressions of *rfaH*, *rfa*, and *rfb* genes in bacterial-resistant community and Δ*rfbC*. Data are shown as log_2_ (fold change). Blank grids indicate undetectable expression. **M**) general structure of lipopolysaccharide in *Salmonella*. Underlined proteins indicate nonsense/frameshift mutations detected in corresponded genes in evolved isolates. **N**) nonsense/frameshift mutations of *rfaH*-regulated *rfb* clusters found in evolved R strains. These strains developed undefeatable resistance and could not be infected by the involved phages (see Fig. 3A). Asterisks represent nonsense mutations, and delta symbols represent frameshift mutations. Dashed arrows indicate the mutation locations. **O**) complemented study of evolved S5–483R1. The *rfaH* gene with a nonsense mutation (Fig. 5N) was replaced by a normal one amplified from WT. The complemented S5–483R1C can be re-infected by SF1 and formed a clear plaque. It also exhibited PR properties after the knock-in.

PR and R isolates evolved from S3 and S5–483 displayed fewer mutations, providing good models for investigating the roles of selected genes associated with resistance development (Fig. 4A). Using S5–483, we successfully generated eight knock-out mutants and their complemented cells. The EOP test showed that mutants, including Δ*sfmH*, Δ*tufB*, Δ*yfiC*, and Δ*pagP*, exhibited similar infectivity compared to the WT (Fig. 5A). In addition, growth suppression tests were conducted to check the roles of selected genes in phage resistance development. The complementation of knockout genes restored the phage growth suppression effect (Fig. S9), confirming the genes' functions in the deletion mutants. Some genes (e.g., *sfmH* and *tufB*) exhibited protective effects against phage treatment because the mutants with deletions of these genes took longer to develop resistance compared to WT cells (Fig. 5B, D). Contrarily, the gene *pagP* played a negative role in resistance development (Fig. 5I). Additionally, the deletion of *mshA* resulted in a lower EOP value (0.1) but did not affect the resistance development (Fig. 5F). In conclusion, although some of these genes may play a role in resistance development, the impact of individual mutations in these genes was limited. In contrast, mutants Δ*rfaH,* Δ*rfaB*, and Δ*rfbC* showed impressive alterations in phage resistance.

The knockout of *rfaH* reduced the EOP by 94.7 folds and conferred robust phage resistance (Fig. 5A, C). It positively regulated the *rfb* and *rfa* gene clusters (Fig. 5M), which determine the O-antigen and LPS core synthesis, respectively [82]. During short-term adaptation (12 h), the expression of *rfaH*, *rfa*, and *rfb* clusters in RC were all suppressed (Fig. 5L). These results indicate a negative correlation between *rfaH* expression and phage resistance development. In contrast, the knockout of *rfaB*, which encodes a galactosyltransferase responsible for adding galactose side groups to the LPS outer core, increased the EOP by 2.3-fold and prevented effective resistance development (Fig. 5A, G, M). This stipulated that the galactose side groups protect host bacteria and reduce phage infectivity. Moreover, the knockout of *rfbC*, a member of the *rfb* cluster, completely prevented plaque formation and bacterial growth inhibition of SF1 (Fig. 5A, E), indicating the development of complete resistance. The loss of dTDP-4-dehydrorhamnose 3,5-epimerase, coded by *rfbC*, blocked the synthesis of dTDP-rhamnose, resulting in a lack of serotype-specific O-antigen in Δ*rfbC* (Fig. 5M). Furthermore, the loss of O-antigen led to significant morphological alterations compared to WT cells: roughness in Δ*rfbC*, and aggregative phenotypes in Δ*rfaH* and Δ*rfbC* (Fig. 5C, E, J). Mutations in *rfaH* and *rfb* cluster genes were recorded in many evolved R isolates (Tables S5, S6, and S7). This also explained the lowered fitness in R strains (Fig. 2J). Further phage adsorption tests showed that the attachment of phage SF1 to Δ*rfaH* and Δ*rfbC* was completely prevented (Fig. 5K). These data further confirmed that the O-antigen is the binding receptor for phage SF1 [83–85].

We further examined the genetic profiles of evolved bacterial mutants in detail and found that many, but not all, R mutants contained mutations in the *rfaH* and *rfb* genes (Fig. 5M, Tables S5, S6, and S7). In addition, all these mutations were either frameshift or nonsense mutations, located at the beginning or middle of *rfaH*/*rfb* genes, indicating the functional loss of coded proteins (Fig. 5N). Furthermore, by comparing with data in Fig. 3A, we found that R strains containing such mutations could not be infected by evolved phages, whereas R strains without *rfaH*/*rfb* mutations could be infected. Thus, we classified the mutations accumulated in evolved bacteria into two classes: key mutations (KMs) and secondary mutations (SMs). KMs, which involve frameshift or nonsense mutations in the *rfaH*-regulated *rfb* cluster genes, led to defects in O-antigen synthesis, making R strains with KMs undefeatable. In contrast, SMs, comprising other mutations contributing to phage resistance but not as critical, led to PR and R strains with defenses that could be breached by evolved phages. To further confirm our theory, a functional complementation test was conducted. The evolved mutant strain S5–483R1, which contains a KM (*rfaH*) and SMs (e.g., *mshA* and *sfmH*), was chosen. The KM gene in S5–483R1 was knocked out, and then a normal *rfaH* gene amplified from WT cells was knocked in. The resulting complementary mutant could be re-infected by phage SF1, indicating the critical role of KM in R development during co-evolution (Fig. 5O).

## Conclusions

In this study, we observed both phage extinction and co-existence after 30 days of host-phage co-evolution. This provides an excellent model to understand the molecular basis leading to different evolutionary outcomes. Firstly, we characterized the phage resistance that developed in host bacteria during co-evolution. Various extracellular and intracellular defense mechanisms against phage infection have been reported [23, 24, 31]. It was intriguing that surface modification was found to dominate the defense during co-evolution in this study. The phage resistance of evolved populations and phage adsorption rates in all three groups exhibited strong linear correlations. Comparable outcomes were observed in another extensive co-evolution study performed in natural environments [86]. Alterations to the cell surface decrease phage adsorption efficiency, facilitating resistance. These passive extracellular defenses are considered more effective than intracellular ones, as they prevent the phage from entering the cell [44]. Moreover, the fitness cost of the R population in the phage extinction group was significantly lower than that of the co-existence groups. This lower cost further accelerated successful resistance development and contributed to the phage extinction [13, 22]. Besides bacterial evolution, we also characterized viral evolution in the co-existence groups. The arms race conferred various phenotypes on evolved phages, such as improved burst size, leading to a better inhibitory effect against the ancestral host. Additionally, some evolved phages were able to infect certain R strains that had blocked the adsorption of the ancestor phage. This capability could be a crucial factor influencing population dynamics. As the microbial community paid a fitness cost to develop unreliable defenses, it became more difficult for the host to eliminate the parasites.

**Figure 6:**
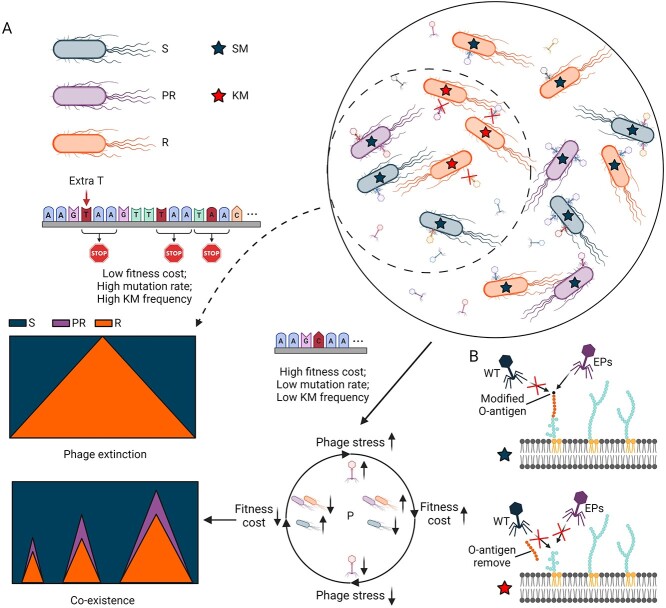
**Model for factors contributing to different evolutionary dynamics and outcomes**. **A**) some host strains exhibit a lower fitness cost to develop resistance quickly (high mutation rate) and strongly (high key mutation frequency) under phage stress (in dashed circle). After a short period of co-evolution, R population dominates the bacterial community and wipes out parasites from the mixed system. Subsequently, the survived S population recovers due to their fitness superiority. For some other strains with defects in these factors mentioned above, they have to co-exist with parasites (in sold circle). The R population of these strains incur a significantly higher fitness cost to develop resistance, leading to fitness advantages in the PR and S populations when the phage stress is released. The population dynamics enter a balanced cycle of phage stress and fitness cost, resulting in fluctuating selection dynamics and eventual co-existence. **B**) key mutations lead to undefeatable phage resistance. During co-evolution, some evolved bacterial populations can develop strong resistance that cannot be infected by evolved phages. They remove the membrane receptor (O-antigen) via nonsense/frameshift mutations in some critical genes, and then stop the phage attachment. S, sensitive; PR, partial resistant; R, completely resistant; SM, secondary mutation; KM, key mutation; P, population; WT, wild-type phage; EP, evolved phage.

Molecular characterization pinpointed genetic changes in both host and phage populations that support the assumption of an arms race co-evolution. Bacterial genetic mutations concentrated in O-antigen synthesis metabolism, while mutations in tail fiber proteins were prevalent in evolved phages. More mutations accumulated in the phage extinction group than in the co-existence groups, indicating a higher chance of developing resistance faster and more effectively [87, 88]. Further gene knock-out experiments demonstrated that the *rfaH*-regulated *rfb* cluster, which involved in O-antigen synthesis, played a critical role in developing resistance [89–91]. Moreover, the R strains containing nonsense/frameshift mutations in *rfaH*/*rfb* could not be infected by evolved phages, whereas the R strains without these mutations could be. Thus, we defined KM and SM in evolved bacterial mutants. SMs might lead to extra/intracellular alterations in host cells, resulting in complete resistance that can be overcome by involved phages. Conversely, the presence of KMs results in receptor removal, leading to undefeatable resistance in bacteria (Fig. 6B).

In conclusion, the outcomes of host-phage co-evolution could be determined by specific characteristics of the host bacteria, that leads to different trade-offs between phage resistance (receptor modification in this study) and fitness cost. In the case of phage extinction, bacteria exhibit lower fitness costs, due to higher mutation rates and frequencies of KMs, and can develop rapid and robust phage resistance. Conversely, bacterial strains displaying higher resistance costs with lower mutation rates and fewer KMs, tend to adopt a co-existence strategy, and they are unable to eradicate the parasites post initial selection rounds. This dynamic results in fluctuating selection and co-existence. Moreover, the balance between phage stress and fitness cost of evolved populations with different resistance phenotypes (S, PR, and R) result in fluctuating selection dynamics in the co-existence model (Fig. 6A). Overall, these findings offer comprehensive insights into the phenotypic and genomic co-evolution of host-phage interactions. They highlight critical factors, such as mutation rates and key mutations for receptor modification, that play a role in the trade-offs between the development of phage resistance and fitness cost, ultimately determining evolutionary outcomes.

## Supplementary Material

Supplementary_Figures_wrae214

Supplementary_Tables_wrae214

## Data Availability

Sequenced bacterial genomes and short-read resequencing data have been deposited under the NCBI BioProject with accession number PRJNA1012466. Genome of phage SF1 can be accessed in NCBI database via MK770409.
