# Supplementary material for: Trade-offs between receptor modification and fitness drive host-bacteriophage co-evolution leading to phage extinction or co-existence
Source: ISME J. 2024 Oct 23;18(1):wrae214. doi: 10.1093/ismejo/wrae214 (PMC11538992; doi:10.1093/ismejo/wrae214)
Supplement: Supplementary_Figures_wrae214 [file supplementary_figures_wrae214.docx]

**Supplementary Figures for**

**Trade-offs between receptor modification and fitness drive host-bacteriophage co-evolution leading to phage extinction or co-existence**

Lin Chen^a, b^, Xue Zhao^c^, Shelyn Wongso^a^, Zhuohui Lin^d^, Siyun Wang^a*^

*^a^ Food, Nutrition and Health, Faculty of Land and Food Systems, The University of British Columbia, Vancouver, British Columbia V6T 1Z4, Canada*

*^b^ School of Chemistry, Chemical Engineering and Biotechnology, Nanyang Technological University, Singapore 637459, Singapore*

*^c^ Department of Biological Systems Engineering, Virginia Tech, Blacksburg, Virginia 24061, United States*

*^d^ Faculty of Microbiology and Immunology, The University of British Columbia, Vancouver, British Columbia V6T 1Z4, Canada*

*^*^* Corresponding author:

Siyun Wang, Ph.D.

Professor of Food Safety Engineering

Food, Nutrition and Health, Faculty of Land and Food Systems

The University of British Columbia

Vancouver, BC Canada, V6T 1Z4

Tel: (+1) 604.827.1734; Fax: (+1) 604.822.6394

Email: siyun.wang@ubc.ca


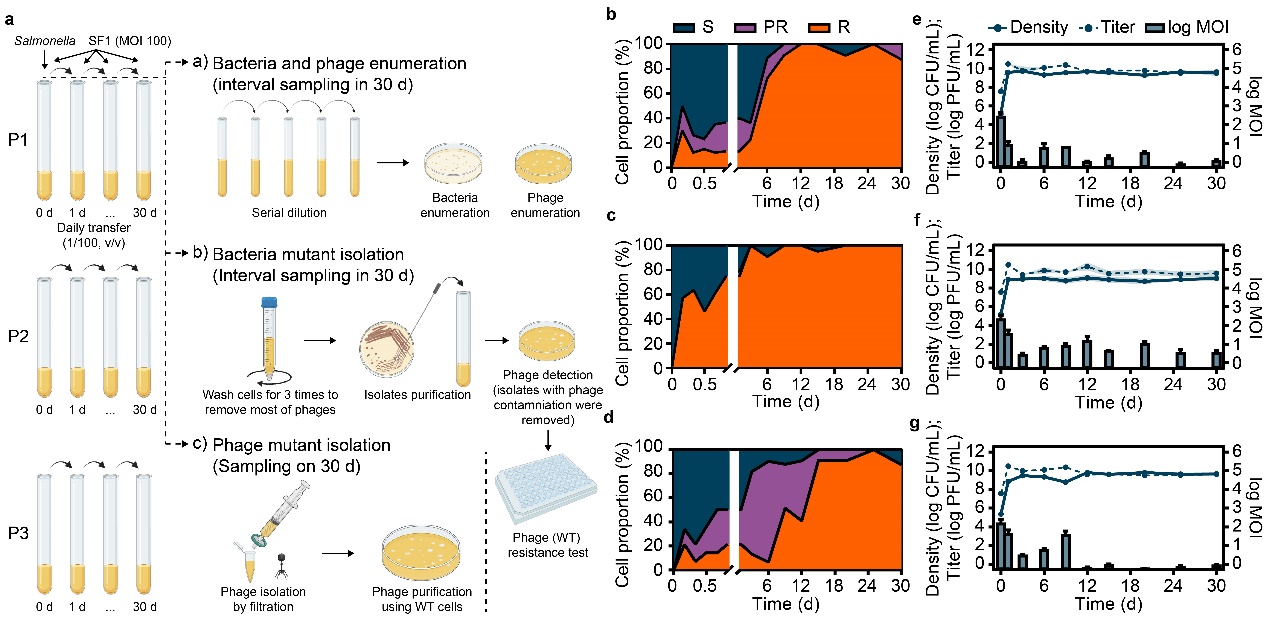


**Fig. S1. Co-evolution of *Salmonella* enteritidis strains with daily supplied *Siphoviridae* phage SF1 (MOI 100). a**, A schematic diagram represented a 30-day co-evolution test. Three *Salmonella* strains (three replicates per strain) were propagated with the lytic phage SF1 and continuously transferred for 30 days. During the daily transfer, SF1 phage (final concentration, 10^9^ PFU/mL) was added to keep the initial MOI at around 100. Figure created with BioRender.com. Proportion changes of sensitive (S), partial resistant (PR) and completely resistant (R) populations in the evolutionary groups of S3 (**b**), S187 (**c**) and S5-483 (**d**). For each group at each timepoint, bacteria-phage mixtures were collected and washed three times to remove most of the phage particles. They were then spread on plates and 45 evolved isolates were purified. Their phage resistance patterns were examined using 96-well plates and a plate reader, using a liquid medium-based assay. Changes of total bacterial density, phage titer, and log MOI in S3 (**e**), S187 (**f**) and S5-483 (**g**) evolution groups. Before daily transferring, bacteria-phage mixtures were sampled, and bacteria enumeration was performed. After filtration, phage titer in medium was measured on infused soft agar. Bacteria density and phage titer were shown by solid and dashed lines, respectively (blue shaded area, mean ± s.d.; n = 3). Log MOI was calculated based on the bacteria density and phage titer at each timepoint and shown by bar graphs. Error bars represent one s.d. above the mean values (n = 3).


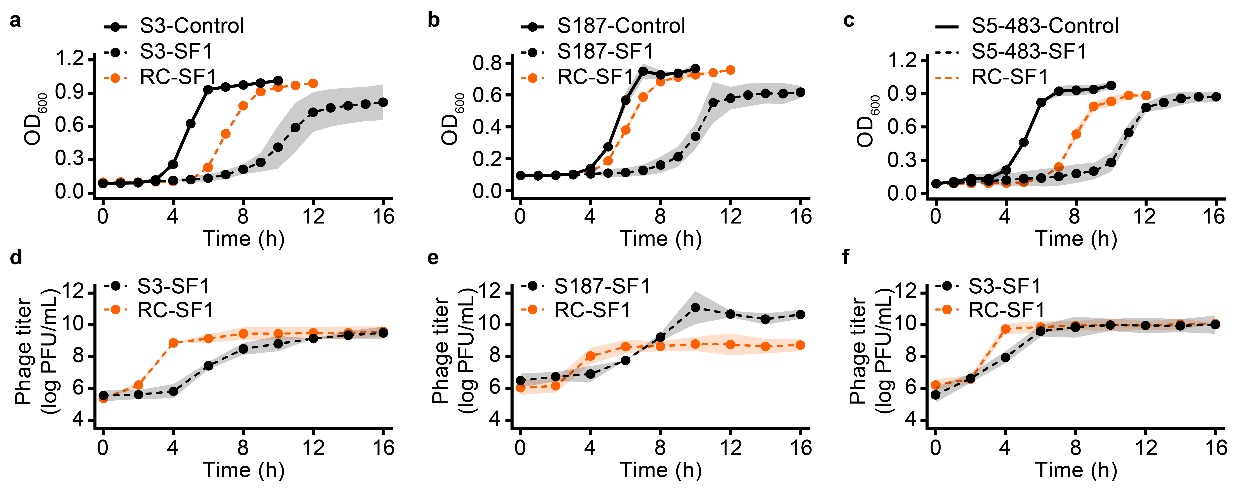


**Fig. S2. The successful development of phage resistance in three *Salmonella* strains.** Growth curves of *S.* enteritidis strains S3 (**a**), S187 (**b**), S5-483 (**c**) and their resistant community (RC) cells under phage SF1 treatment at MOI 100. RC cells were collected from phage SF1-treated cells grown to early stationary phase (around 12 h). They were washed three time to remove the most of phage particles, and then diluted to around 10^5^ CFU/mL, mixed with phage at MOI 100 and cultured in 96-well plates at 37℃ for 16 h. The dynamics of phage titers in the co-culture medium of S3 (**d**), S187 (**e**), S5-483 (**f**), and their RC cells were measured. Bacteria density/phage titer dynamics were shown by solid (WT cells without phage treatment) and dashed lines (black, WT cells with phage treatment; orange, RC cells with phage treatment) during 16 h of co-culture (shaded area, mean ± s.d.; n = 3).


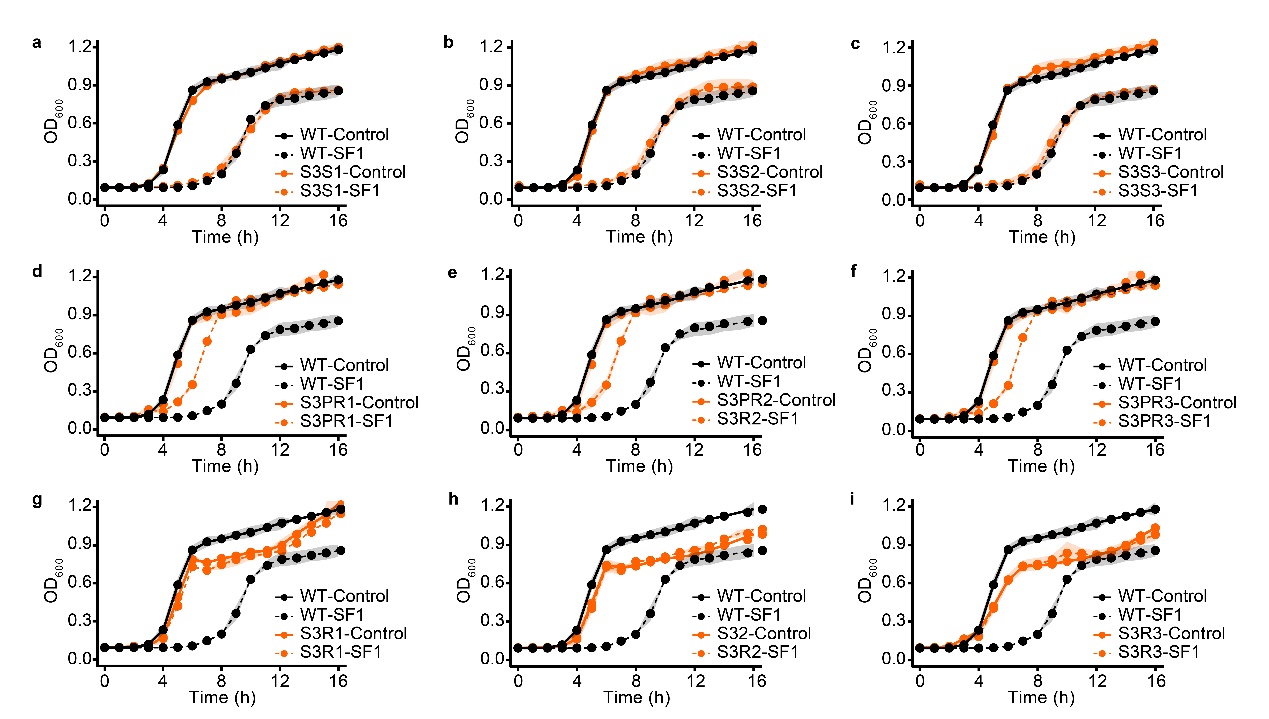


**Fig. S3. Growth curves of WT and isolates evolved from S3 under phage SF1 treatment at MOI 100.** Representative evolved isolates were labeled as ancestor strain, evolved resistant pattern and isolate number. An isolate was defined as sensitive (S) if it exhibited the same cell density dynamics as WT cells in the presence of phage, and completely resistant (R) if it showed uninhibited growth. The partial resistant (PR) pattern was characterized by cells displaying less growth inhibition compared to WT cells under phage treatment. They were selected at the latest timepoint at which resistance was detected, from each replicative population (see Fig. 1a). Sold lines in black and orange are growth curves of WT and evolved isolate, respectively, without phage treatment (control). Dashed lines in black and orange are growth curves of WT and evolved isolate, respectively, under phage treatment (shaded area, mean ± s.d.; n = 3).


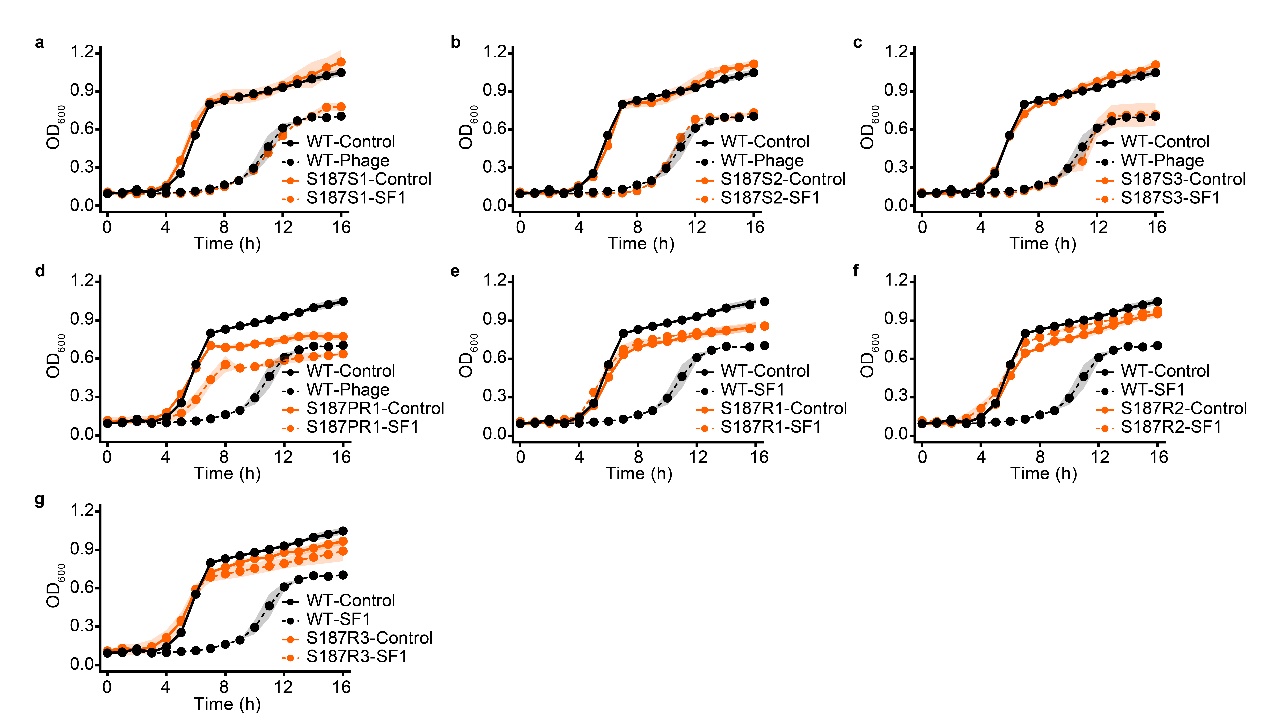


**Fig. S4. Growth curves of WT and isolates evolved from S187 under phage SF1 treatment at MOI 100.** Isolates were labeled, classified and selected as in Fig. S3. Sold lines in black and orange are growth curves of WT and evolved isolate, respectively, without phage treatment (control). Dashed lines in black and orange are growth curves of WT and evolved isolate, respectively, under phage treatment (shaded area, mean ± s.d.; n = 3).


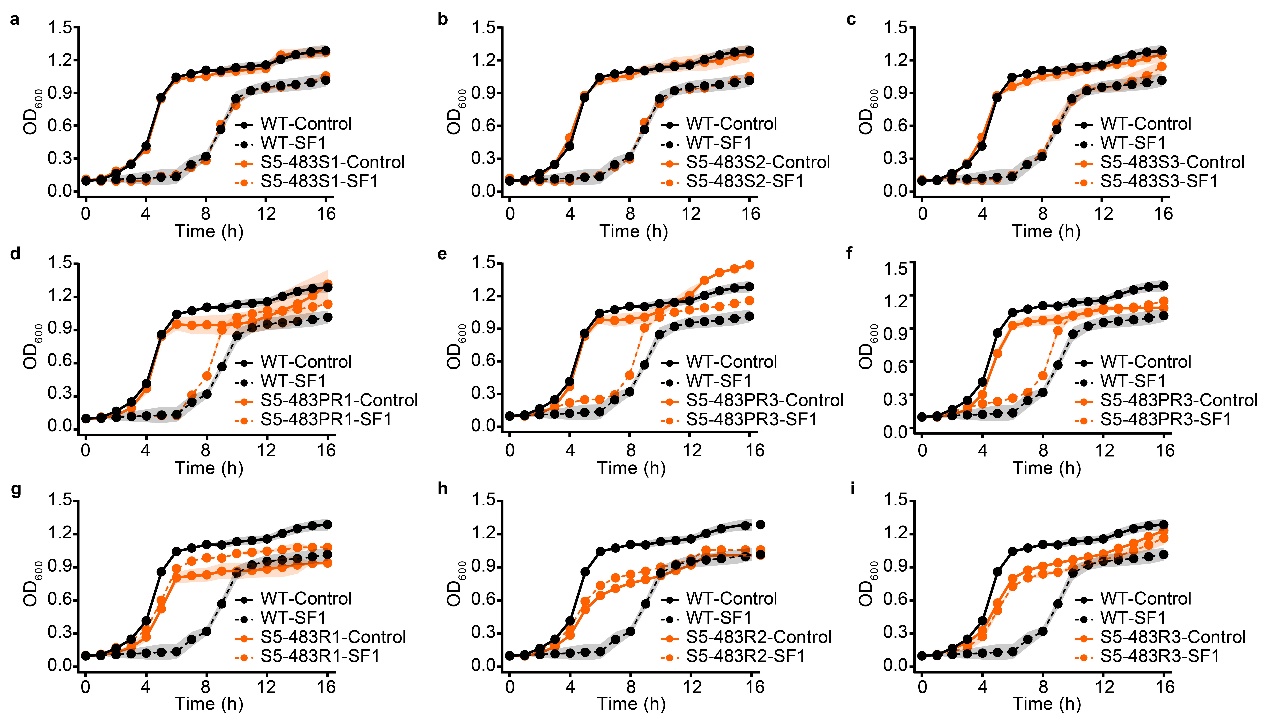


**Fig. S5. Growth curves of WT and isolates evolved from S5-483 under phage SF1 treatment at MOI 100.** Isolates were labeled, classified and selected as in Fig. S3. Sold lines in black and orange are growth curves of WT and evolved isolate, respectively, without phage treatment (control). Dashed lines in black and orange are growth curves of WT and evolved isolate, respectively, under phage treatment (shaded area, mean ± s.d.; n = 3).


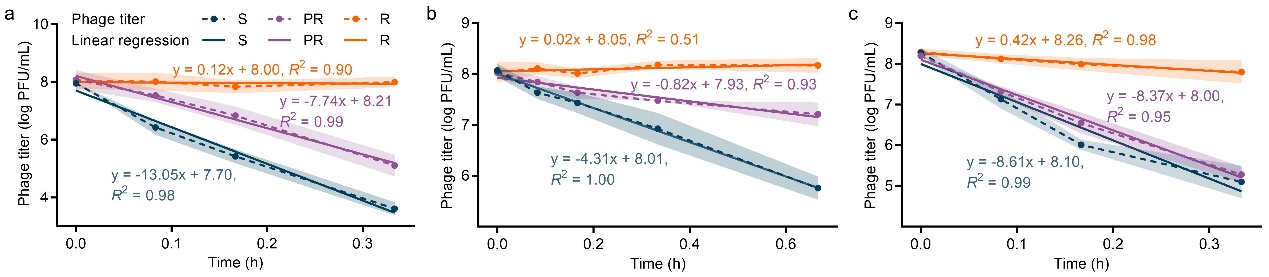


**Fig. S6. Linear regression of phage adsorption dynamics. a-c,** Linear regression of phage adsorption dynamics of isolates with different phage resistance patterns (S, sensitive; PR, partial resistant; R, completely resistant). Isolates were mixed with SF1 phage at MOI 5 for 20 (S3 and S5-483 groups) or 40 min (S187 group) before burst. They evolved from S3 (**a**), S187 (**b**), and S5-483 (**c**), respectively. Representative S, PR, and R isolates were selected from each replicative population (except PR isolate evolved from S187, only one strain was isolated). Shaded areas indicated 95% confidence.


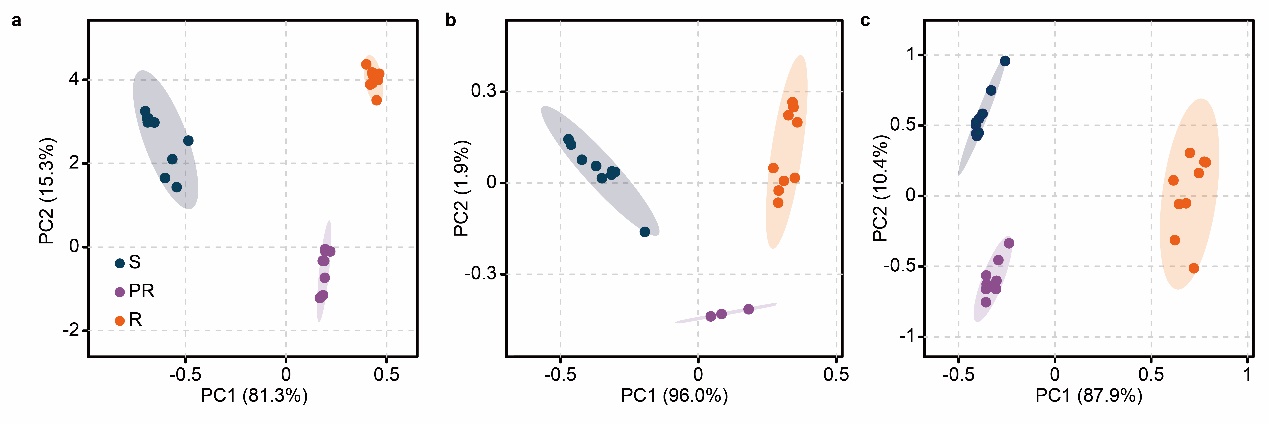


**Fig. S7. Development of phage resistance and related fitness cost. a**-**c**, Principal component analysis (PCA) of isolates with different phage resistance patterns (S, sensitive; PR, partial resistant; R, completely resistant). Isolates evolved from S3 (**a**), S187 (**b**), and S5-483 (**c**), respectively. Growth curves of evolved isolates (see Fig. S3, Fig. S4 and Fig. S5) under phage treatment were subject to PCA. Representative S, PR and R isolates were selected from each replicative population (except PR isolate evolved from S187, only one strain was isolated). Shaded areas indicated 95% confidence.


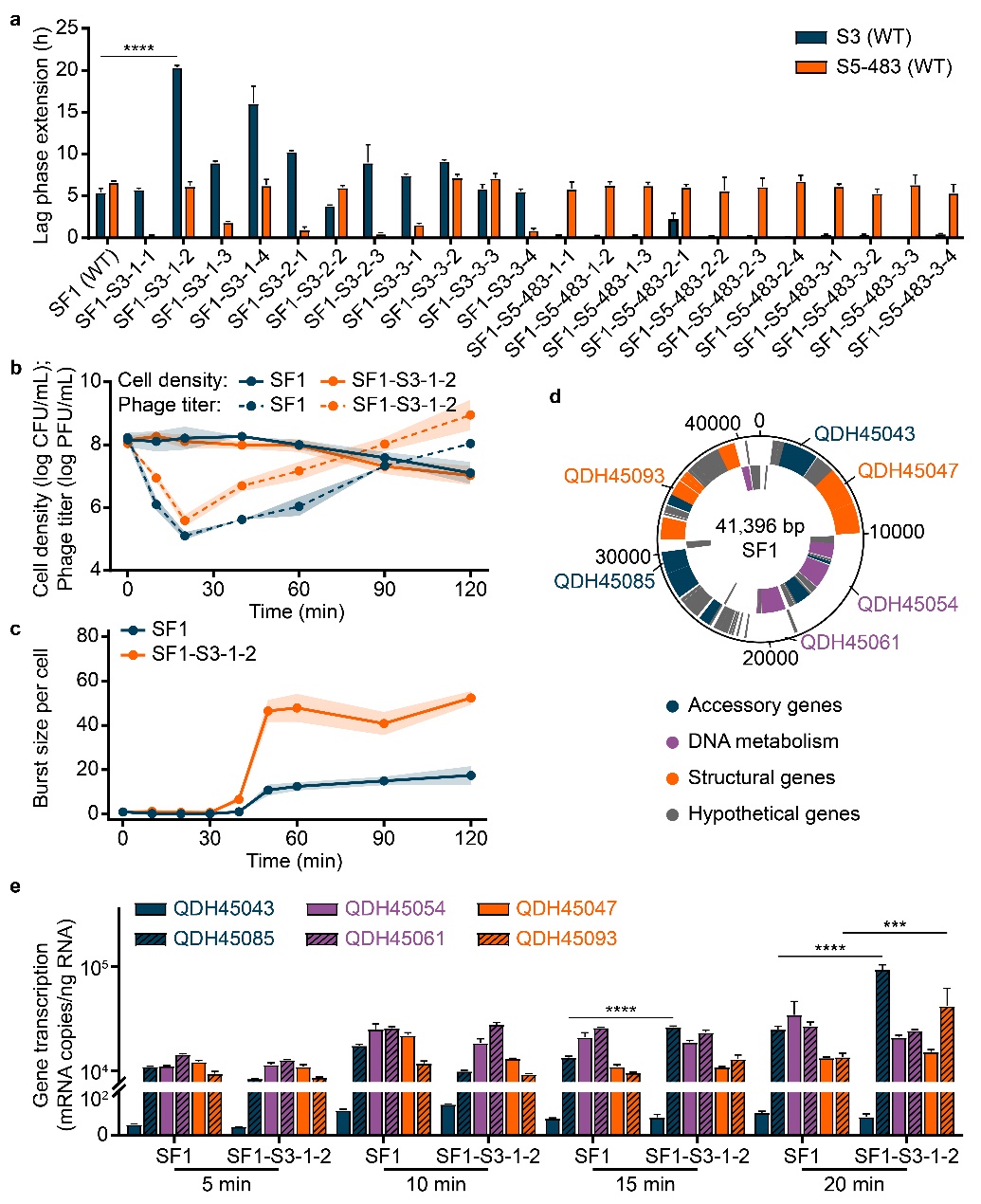


**Fig. S8. Evolved phages with better inhibitive effects on bacterial growth.** **a**, Lag phase extension of evolved phages against WT S3 and S5-483 strains. Data are representative of three biological replicates and the error bars above represent one s.d. Asterisks above bars indicate significant pairwise differences using one-way ANOVA, post hoc LSD test (*****P* < 0.0001). **b**, Attachment dynamics of ancestor SF1 and evolved SF1-S3-1-2 onto WT S3 cells. Bacteria density and phage titer during attachment were shown by solid and dashed lines, respectively (shaded area, mean ± s.d.; n = 3). **c**, One-step growth curve of SF1 and SF1-S3-1-2. S3 cells were mixed with phages at MOI 0.1. After adsorption for 10 min, excess phages were removed by centrifugation, and the titers of released phage were then measured at intervals (shaded area, mean ± s.d.; n = 3). **d**, SF1 genome; genes on the positive (outer circle) or negative (inner circle) strand are indicated. Access no. (NCBI) of proteins coded by selected genes were labeled. Structural genes (orange), DNA metabolism (purple), accessory genes (blue) and hypothetical genes (grey) were selected. **e**, Transcript abundance of selected phage genes during infection (first 20 min). Data are graphed as mean ± s.d., n = 3. Asterisks above bars indicate significant pairwise differences using one-way ANOVA, post hoc LSD test (****P* < 0.001, *****P* < 0.0001).


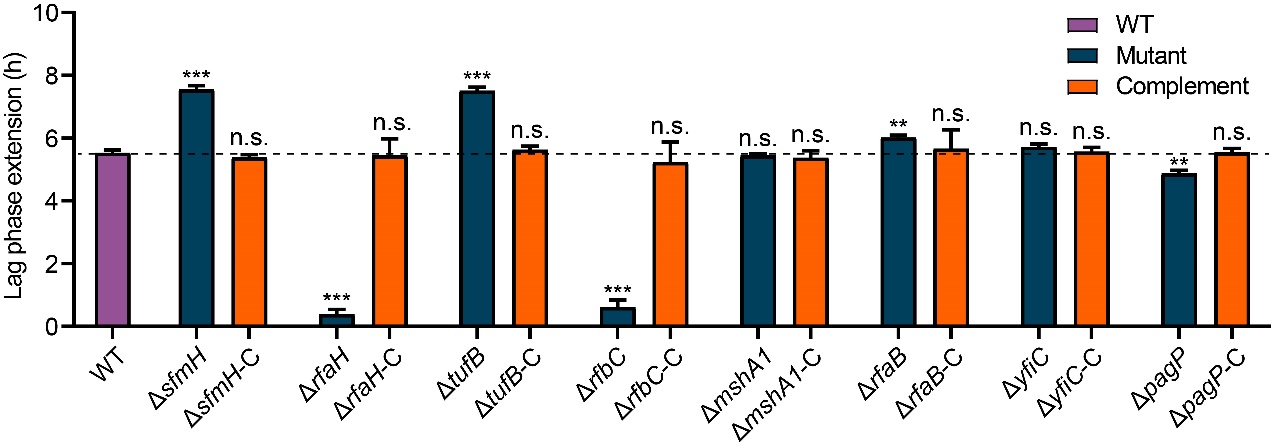


**Fig. S9.** Lag phase extension of wild-type (WT), mutants, and complemented cells under phage SF1 treatment. Data are representative of three biological replicates and the error bars above represent one s.d. Asterisks above bars indicate significant pairwise differences with WT group using one-way ANOVA, post hoc LSD test (n.s. *P* > 0.05, ***P* < 0.01, ****P* < 0.001).
